# Limited impact of a nudge comment in promoting de-escalation to first-generation cephalosporins in urinary tract infections

**DOI:** 10.1017/ash.2022.297

**Published:** 2022-09-26

**Authors:** Martin T. Brenneman, Sarah E. Moore, Matthew Song, Brian C. Bohn, Elena A. Swingler, Christopher W. Whitman, Alan D. Junkins, Ashley M. Wilde

**Affiliations:** 1 Pharmacy Department, Norton Healthcare, Louisville, Kentucky; 2 Norton Infectious Diseases Institute, Norton Healthcare, Louisville, Kentucky; 3 Department of Pharmacy, Barnes-Jewish Hospital, Saint Louis, Missouri; 4 Pharmacy Department, Mobile Infirmary Medical Center, Mobile, Alabama; 5 Microbiology Department, Norton Healthcare, Louisville, Kentucky

## Abstract

In this study, we evaluated the impact of a microbiology nudge on de-escalation to first-generation cephalosporins in hospitalized patients with urinary tract infections secondary to *Escherichia coli*, *Klebsiella pneumoniae*, and *Proteus mirabilis* isolates with minimum inhibitory concentrations (MICs) ≤ 16 µg/mL. De-escalation to first generation-cephalosporins was uncommon at MICs = 4–16 µg/mL.

Urinary tract infections (UTIs) are among the most common bacterial infections in the United States.^1^ Many antimicrobials achieve higher concentrations in the urine than at other body sites.^2^ The Clinical and Laboratory Standards Institute designated a cefazolin minimum inhibitory concentration (MIC) ≤ 16 µg/mL as susceptible for parenteral cefazolin and oral cephalosporins for the treatment of uncomplicated UTIs caused by *Escherichia coli*, *Klebsiella pneumoniae*, and *Proteus mirabilis*. This break point is higher than MIC ≤ 2 µg/mL, which is used for other infections.^3^

Antimicrobial stewardship nudges are nonrestricting with low resource burden.^4–6^ Nudges have reduced inappropriate treatment of asymptomatic bacteriuria, decreased broad-spectrum antibiotic use, and increased collection of blood cultures after isolating *Staphylococcus aureus* from urine cultures.^7–9^ These nudges suggested a specific action or noted the presence or absence of a bacteria or symptomatology. Our nudge promoted the linkage of 2 pieces of information to guide decision making: syndrome and MIC. We investigated whether the microbiology nudge promoted de-escalation to first-generation cephalosporins across all MICs ≤ 16 µg/mL.

## Methods

This retrospective cohort study included hospitalized patients treated for UTIs between January 1, 2020, and May 31, 2021. It was conducted at Norton Healthcare, a community healthcare system in Louisville, Kentucky, with 4 adult hospitals and >1,500 licensed beds. The system employs 4 infectious diseases (ID) pharmacists and 1 ID pharmacy resident. The University of Louisville Institutional Review Board approved this study with a waiver of informed consent.

In October 2019, our microbiology laboratory implemented antimicrobial susceptibility testing (AST) panels that included doubling cefazolin dilutions between 2 and 16 µg/mL. The AST reports within the electronic medical record began reporting actual cefazolin MIC; however, interpretations were reported as susceptible (MIC ≤ 2 µg/mL), intermediate (MIC = 4 µg/mL), or resistant (MIC ≥ 8 µg/mL). A nudge was added to urine culture AST reports for appropriate organisms with cefazolin MICs of 2–16 µg/mL, which read as follows: “Isolates with cefazolin MICs ≤16 remain susceptible to oral cephalosporins for the treatment of uncomplicated urinary tract infections.” Local treatment guidelines already recommended first-generation cephalosporins as first-line treatment for susceptible Enterobacterales. In-person and e-mail education of hospitalists was completed prior to the study period; this group admits most patients in our system.

Included patients were aged ≥18 years, had been admitted to an adult hospital, and had a urine culture with *Escherichia coli*, *Klebsiella pneumoniae*, or *Proteus mirabilis* isolated. Additional inclusion criteria were receipt of at least 1 dose of ceftriaxone, ceftazidime, cefepime, ciprofloxacin, levofloxacin, piperacillin-tazobactam, or meropenem indicated for a UTI administered within 48 hours of urine-culture collection. These antibiotics require indication selection on order entry and are flagged for prospective audit and feedback by antimicrobial stewardship program (ASP) staff. Patients were included once. Exclusion criteria were polymicrobial urine culture and hospital discharge prior to or within 4 hours of finalized AST results. Patients were grouped by urine isolate cefazolin MIC determined using MicroScan (Beckman Coulter, Brea CA). The cohorts were defined as follows: fully susceptible (MIC ≤2 µg/mL), intermediate (MIC = 4 µg/mL), UTI susceptible (MIC 8–16 µg/mL), and fully resistant (MIC >16 µg/mL).

The primary outcome was percentage of patients prescribed a first-generation cephalosporin (cephalexin or cefazolin) after urine-culture AST results were obtained. Secondary outcomes included percentage of patients with a recommendation to de-escalate to first-generation cephalosporins and a comparison of the primary outcome between patients with and without a recommendation from the ASP. ASP recommendations are documented in the electronic health record as part of normal workflow.

All records of patients from the intermediate cohort (MIC = 4 µg/mL) and the UTI-susceptible cohort (MIC = 8–16 µg/mL) were reviewed for potential barriers to de-escalation. These barriers included cephalosporin allergy, secondary indication for antibiotics, intensive care unit (ICU) admission during hospitalization, bacteremia, invasive urologic procedures during or within 14 days of hospitalization (defined as cystoscopy, cystectomy, renal stent placement, nephrostomy placement or removal, transurethral surgery of the prostate or bladder, urethroscopy, or percutaneous stone surgery), and complicating factors (including pyelonephritis, renal calculi, obstructions, chronic urinary catheter use, and neurogenic bladder). Additionally, data regarding susceptibility to fluoroquinolones, nitrofurantoin, and trimethoprim-sulfamethoxazole were collected.

Continuous and categorical variables were summarized with descriptive statistics. Between group comparisons for continuous and categorical variables were made using the Kruskal-Wallis and χ^2^ tests, respectively. Outcomes were assessed with Cochran-Armitage test for trend. Multiple logistic regression was used to identify relationships between covariates and rate of de-escalation.

## Results

We included 1,387 patients in this study, and 203 patients (14.6%) were de-escalated to a first-generation cephalosporin. Baseline characteristics for all patients can be found in Table 1. First-generation cephalosporins were utilized in 21% of the fully susceptible cohort, in 4% of the intermediate cohort, and in 2% of the UTI susceptible cohort (Table 1). Test for trend identified a significant decrease in the rate of de-escalation as cefazolin MIC increased (*P* < .001).

**Table 1.** Baseline characteristics and outcomes

Urine Isolate Cohorts	Fully susceptible (MIC ≤2 μg/mL)	Intermediate (MIC 4 μg/mL)	UTI susceptible (MIC 8-16 μg/mL)	Fully resistant (MIC 16 μg/mL)	p-value
	n = 924	n = 141	n = 90	n = 232	
**Age, median [IQR]**	71 [57, 80]	71 [56, 80]	73 [59, 81]	70 [57, 78]	0.642
**Female**	753 (81)	108 (77)	69 (77)	171 (74)	**0.043**
**LOS, days (median [IQR])**	4.70 [3.15, 7.18]	4.59 [3.29, 6.77]	4.60 [3.16, 8.25]	5.55 [3.90, 9.01]	**0.001**
**Urine culture organisms**					**<0.001**
*Escherichia coli*	647 (70)	123 (87)	76 (84)	198 (85)	
*Klebsiella pneumoniae*	212 (23)	3 (2)	6 (7)	25 (11)	
*Proteus mirabilis*	65 (7)	15 (11)	8 (9)	9 (4)	
**Empiric antibiotic name**					**<0.001**
ceftriaxone	810 (88)	121 (86)	79 (88)	173 (75)	
ceftazidime	1 (<1)	0 (0)	0 (0)	0 (0)	
cefepime	51 (6)	10 (7)	5 (6)	28 (12)	
ciprofloxacin	14 (2)	0 (0)	2 (2)	1 (<1)	
levofloxacin	17 (2)	6 (4)	1 (1)	3 (1)	
piperacillin/tazobactam	29 (3)	4 (3)	3 (3)	22 (9)	
meropenem	2 (<1)	0 (0)	0 (0)	5 (2)	
**Primary outcome**					
De-escalated to first-generation cephalosporin	195 (21)	5 (4)	2 (2)	1 (<1)	**<0.001**
**Secondary outcomes**					
Recommendation made to de-escalate to a first-generation cephalosporin	108 (12)	3 (2)	0 (0)	1 (<1)	**<0.001**
De-escalated with ASP intervention	50 (5)	1 (1)	0 (0)	0 (0)	**<0.001**
De-escalated without ASP intervention	145 (16)	4 (3)	2 (2)	1 (<1)	**<0.001**

All values reported as n (%) unless otherwise noted, ASP = Antimicrobial stewardship program, AST = antimicrobial susceptibility testing, LOS = Length of stay, MIC = minimum inhibitory concentration, UTI = urinary tract infection.

The ASP recommended de-escalation to first-generation cephalosporins in 112 (8%) of all patients; 51 recommendations (46%) were accepted. Only 1 recommendation to de-escalate was accepted when the pathogen MIC was >2 μg/mL. As cefazolin MIC increased, test for trend identified significant decreases in the rate of ASP recommendations made (*P* < .001) and the rate of de-escalations (Table 1).

In the subgroup of patients with isolates that had MICs 4–16 μg/mL, 3% of patients were de-escalated to a first-generation cephalosporin. Statistical tests were not performed due to sample size constraints. Frequency of complicating factors can be found in Table 2.

**Table 2.** Patients in the intermediate and UTI susceptible cohorts by de-escalation status

	Intermediate or UTI Susceptible	
	Patients not de-escalated to first-generation cephalosporin	Patients de-escalated to first-generation cephalosporin
N	224	7
**Age, median [IQR]**	72 [58, 80]	78 [59, 84]
**Female, n (%)**	173 (77)	4 (57)
**Cefazolin MIC, n (%)**		
4 µg/mL	136 (61)	5 (71)
8 or 16 µg/mL	88 (39)	2 (29)
**History of cephalosporin allergy, n (%)**	14 (6)	0 (0)
**Secondary indication for antibiotics, n (%)**	26 (12)	1 (14)
**Admission to intensive care unit, n (%)**	43 (19)	0 (0)
**Complicating factors of UTI, n (%)**		
Any complicating factor*	86 (38)	1 (14)
Pyelonephritis	35 (16)	0 (0)
Kidney stones	36 (16)	0 (0)
Urinary obstruction	15 (7)	0 (0)
Neurogenic bladder	3 (1)	0 (0)
Urologic procedure	18 (8)	0 (0)
Chronic catheter	13 (6)	0 (0)
Bacteremia	32 (14)	1 (14)
**Urine culture isolate AST data, n (%)**		
Susceptible to fluoroquinolones	134 (60)	4 (57)
Susceptible to nitrofurantoin	187 (84)	5 (71)
Susceptible to TMP/SMX	115 (51)	2 (29)

* Patients could have more than one complicating factor.

ADR = adverse drug reaction, AST = antimicrobial susceptibility testing, IQR = Inter-quartile range.

MIC = minimum inhibitory concentration, TMP/SMX = trimethoprim/sulfamethoxazole, UTI = urinary tract infection.

## Discussion

Despite the nudge comment, lower rates of de-escalation to first-generation cephalosporins at higher MICs were observed. Complicating factors were observed in 38% of those not de-escalated in the subgroup (ie, MICs of 4–16 μg/mL) versus 14% of those who were de-escalated, which suggests these clinical factors contributed to lack of de-escalation. Furthermore, the infrequency of ASP recommendations suggests that de-escalation to a first-generation cephalosporin may not have been considered appropriate by both the prescriber and an ASP pharmacist. Utility of the urinary cephalosporin breakpoint may be lower in an inpatient setting because hospitalized patients are more likely to have complicating factors. Additionally, susceptibility to alternative antimicrobials was reasonably high (51%–84%) in patients not de-escalated to a first-generation cephalosporin, so it is possible that agents listed as susceptible were utilized instead.

Practical barriers with this nudge exist. The nudge required clinicians to correlate syndrome and MIC to take the desired action, rather than providing direct instructions, which may have been a barrier to de-escalation. Our comment was a footnote within the microbiology report and could have simply been overlooked.

This study had several limitations. We only evaluated de-escalation of first-generation cephalosporins. We did not evaluate all oral cephalosporins for which cefazolin is the surrogate, which may have contributed to a higher de-escalation rate. Infrequent de-escalations in the subgroup prevented statistical analysis. Additionally, retrospective review of recommendations may not elucidate the full clinical picture, given that first-generation cephalosporin indication was not collected and unlike empiric therapy, was not limited to UTI. For example, first-generation cephalosporins may have been prescribed for secondary or alternative sources of infection because UTIs are often ruled out after empiric antibiotics have been prescribed. Last, de-escalation to alternative agents and discontinuation were not assessed because these were not actions encouraged by the nudge and may have been the most optimal interventions.

This nudge comment appeared to have minimal impact in promoting first-generation cephalosporin use for urinary tract infections at cefazolin MICs of 4–16 µg/mL. Alternative approaches to promote de-escalation and application in more specific patient populations may warrant further exploration.
